# Regulation of NANOG and SOX2 expression by activin A and a canonical WNT agonist in bovine embryonic stem cells and blastocysts

**DOI:** 10.1242/bio.058669

**Published:** 2021-11-29

**Authors:** Yao Xiao, Froylan Sosa, Pablo J. Ross, Kenneth E. Diffenderfer, Peter J. Hansen

**Affiliations:** 1 Shandong Provincial Key Laboratory of Animal Disease Control and Breeding, Institute of Animal Science and Veterinary Medicine, Shandong Academy of Agricultural Sciences, Jinan, Shandong 250100, China; 2 Department of Animal Sciences, D.H. Barron Reproductive and Perinatal Biology Research Program, and Genetics Institute, University of Florida, Gainesville, FL 32611-0910, USA; 3 Department of Animal Science, University of California, Davis, CA 95616, USA; 4 Stem Cell Core, Salk Institute for Biological Studies, La Jolla, CA 92037, USA

**Keywords:** Embryonic stem cells, Pluripotency, Activin, Bovine, WNT signaling

## Abstract

Bovine embryonic stem cells (ESC) have features associated with the primed pluripotent state including low expression of one of the core pluripotency transcription factors, NANOG. It has been reported that NANOG expression can be upregulated in porcine ESC by treatment with activin A and the WNT agonist CHIR99021. Accordingly, it was tested whether expression of NANOG and another pluripotency factor SOX2 could be stimulated by activin A and the WNT agonist CHIR99021. Immunoreactive NANOG and SOX2 were analyzed for bovine ESC lines derived under conditions in which activin A and CHIR99021 were added singly or in combination. Activin A enhanced NANOG expression but also reduced SOX2 expression. CHIR99021 depressed expression of both NANOG and SOX2. In a second experiment, activin A enhanced blastocyst development while CHIR99021 treatment impaired blastocyst formation and reduced number of blastomeres. Activin A treatment decreased blastomeres in the blastocyst that were positive for either NANOG or SOX2 but increased those that were CDX2^+^ and that were GATA6^+^ outside the inner cell mass. CHIR99021 reduced SOX2^+^ and NANOG^+^ blastomeres without affecting the number or percent of blastomeres that were CDX2^+^ and GATA6^+^. Results indicate activation of activin A signaling stimulates NANOG expression during self-renewal of bovine ESC but suppresses cells expressing pluripotency markers in the blastocyst and increases cells expressing CDX2. Actions of activin A to promote blastocyst development may involve its role in promoting trophectoderm formation. Furthermore, results demonstrate the negative role of canonical WNT signaling in cattle for pluripotency marker expression in ESC and in formation of the inner cell mass and epiblast during embryonic development.

This article has an associated First Person interview with the first author of the paper.

## Abstract (precis)

**Summary:** Activin A (NANOG) and canonical WNT signaling (NANOG and SOX2) inhibit expression of pluripotency factors in bovine embryonic stem cells; activin A also inhibits formation of cells positive for NANOG and SOX2 in the blastocyst while promoting TE formation.

## INTRODUCTION

Embryonic stem cells (ESC) derived from the inner cell mass (ICM) of an early embryo provide excellent models for studying pluripotency and lineage differentiation *in vitro* (Morgani et al., 2017; Watts et al., 2018; Baillie-Benson et al., 2020). Such opportunities were inaccessible in livestock species until recently because culture conditions were inappropriate for stable maintenance of pluripotent characteristics (Navarro et al., 2020). Now, however, culture systems have been developed for culture of ESC from the cow (Bogliotti et al., 2018; Zhao et al., 2021), pig (Choi et al., 2019; Gao et al., 2019), sheep (Vilarino et al., 2020) and horse (Yu et al., 2021). The ESC derived from these species have exhibited robust growth, stable karyotypes, pluripotency-associated marker expression and competent teratoma formation but exhibit various degrees of pluripotency.

Four distinct pluripotent states of ESC have been identified, listed in order of decreasing pluripotency as expanded/extended, naïve, intermediate/formative, and primed pluripotency (Smith, 2017; Yang et al., 2017a,b; Yang et al., 2019). Culture systems for ESC from cattle, pigs and sheep rely on use of fibroblast growth factor 2 (FGF2) and inhibition of WNT signaling. These two conditions are a common feature of systems for derivation of primed pluripotent cells from mouse and human embryos (Wu et al., 2015; Weinberger et al., 2016). Morphological, transcriptomic and epigenomic features of bovine ESC (bESC), including low expression of *NANOG*, have pointed to the cells being in a primed state (Bogliotti et al., 2018).

NANOG, POU5F1 and SOX2 are the core pluripotency transcription factors supporting ESC self-renewal (Chen et al., 2008; Jaenisch and Young, 2008). Deletion of *NANOG* impaired epiblast formation in ICM (Mitsui et al., 2003; Ortega et al., 2020). Although Nanog is dispensable for maintaining pluripotency in mouse ESC, deletion of *Nanog* made mouse ESC prone to commitment signals (Chambers et al., 2007). Elevation of Nanog was associated with the process of resetting epiblast stem cells from a primed to naïve state (Illich et al., 2016; Zhang et al., 2016). Thus, activation of NANOG expression in primed bESC might promote a more pluripotent phenotype.

There are several signals that are responsible for activating *NANOG* expression, including activin A and WNTs (Chatterjee et al., 2015; Vallier et al., 2009; Yi et al., 2011). Activin A controls expression of *NANOG* via activation of the SMAD2/3 cascade (Vallier et al., 2009). In mice, canonical WNT signaling stimulates expression of the three core pluripotency transcription factors through regulation of TCF1/3 activity (Chatterjee et al., 2015; Yi et al., 2011). In the mouse, addition of activin A and Wnt3a enhanced immunoreactive Nanog in epiblast stem cells (Yu et al., 2021). Similarly, in the pig, addition of activin A and the WNT agonist CHIR99021 to culture medium caused ESC to express NANOG at a comparable level to other core pluripotency transcription factors (Choi et al., 2019).

In the current study, we tested the hypothesis that NANOG expression of bESC, which is ordinarily low (Bogliotti et al., 2018), could be increased by the combination of activin A and CHIR99021 while maintaining expression of another core pluripotency transcription factor SOX2. Protein levels of NANOG and SOX2 were analyzed for bESC derived under conditions in which activin A and CHIR99021 were added singly or in combination. Given that cells used to produce bESC are derived from the ICM of the blastocyst, another objective was to test actions of activin A and CHIR99021 on abundance of blastomeres positive for NANOG and SOX2 in blastocyst ICM. Results confirm that activin A can enhance NANOG expression in bESC but also reduce SOX2 expression. Moreover, the major action of activin A on the developing embryo is to promote trophectoderm (TE) while decreasing number of cells positive for NANOG or SOX2. Results also confirm the inhibitory actions of canonical WNT signaling for establishment and maintenance of pluripotency in both bESC and the blastocyst. In particular, CHIR99021 depressed expression of both NANOG and SOX2 in bESC and decreased number and percent of NANOG^+^ and SOX2^+^ blastomeres in the blastocyst.

## RESULTS

### Actions of activin A and canonical WNT signaling on expression of NANOG and SOX2 in bovine embryonic stem cells

**Fig. 1.**
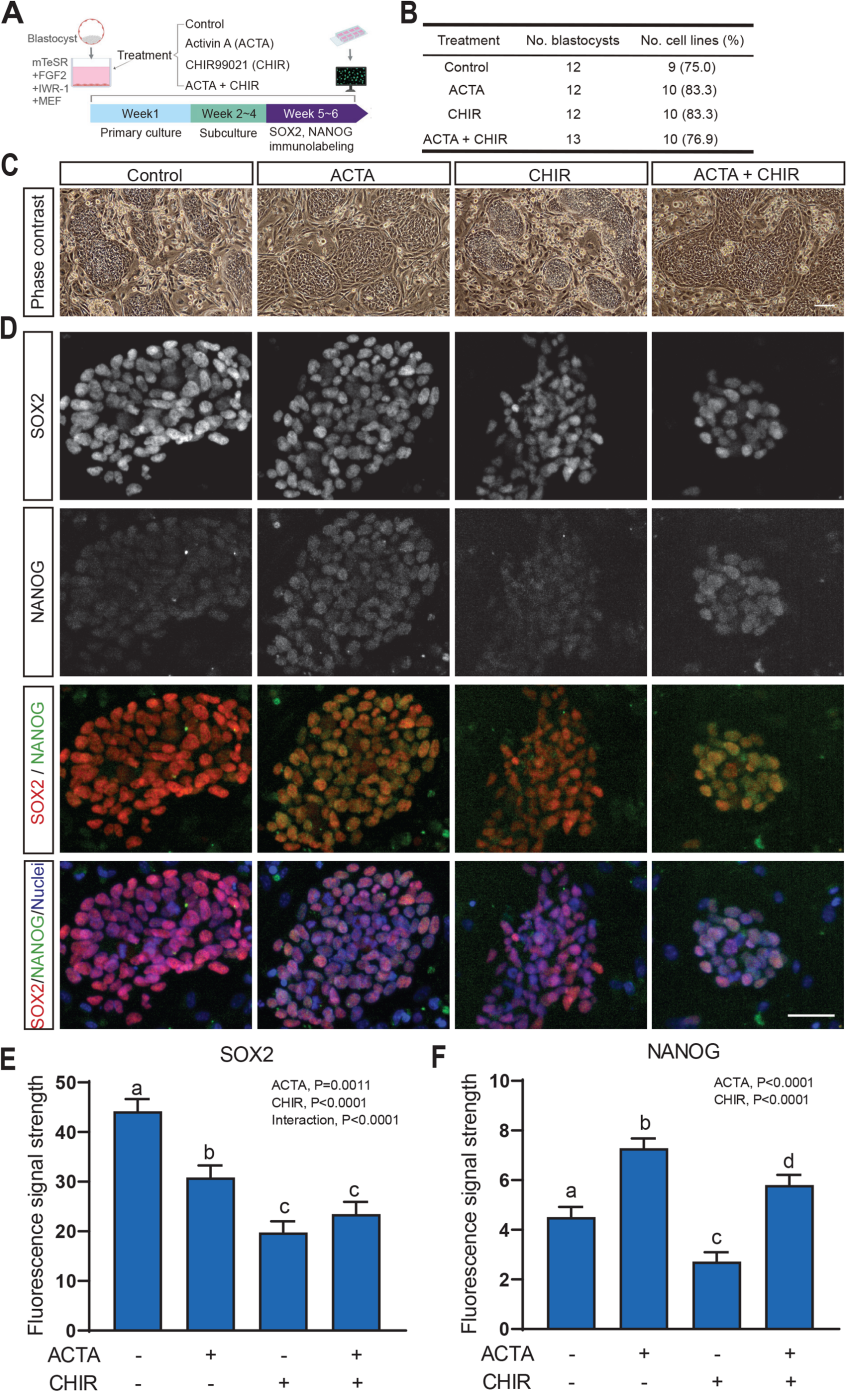
**Treatment of embryonic stem cells with activin A enhanced NANOG expression while activation of canonical WNT signaling attenuated SOX2 and NANOG expression.** (A) Experimental design. Zona-free blastocysts were seeded onto mouse embryonic fibroblasts (MEF) with the culture medium consisting of base medium mTeSR, FGF2, IWR-1 and additional treatments of 25 ng/ml activin A (ACTA), 1.5 μM CHIR99021 (CHIR), 25 ng/ml ACTA+1.5 μM CHIR or control. The total number of blastocysts analyzed is in panel B. Blastocysts were produced in two embryo production replicates. (B) Efficiency of derivation of cell lines. There was no significant difference among treatments. Subsequent observations were carried out on the number of cell lines indicated. (C) Morphological characteristics of cells derived under different conditions at week 5 of passage 5 or 6. Scale bar: 100 μm. (D) Dual immunolabeling of SOX2 and NANOG after 5–6 weeks of treatment. Scale bar: 50 μm. (E, F) Quantification of immunofluorescence for SOX2 (E) and NANOG (F). Data are least-squares means±SEM. The *P*-value for main effects and the interaction that were *P*<0.10 or less are shown in the upper left regions of each figure. Bars with different letters differ (*P*<0.05) as determined by mean-separation test.

The hypothesis that addition of activin A, the WNT agonist CHIR99021 or both would enhance NANOG expression was tested. Cell lines were derived from blastocysts using 20 ng/ml FGF2, 2.5 μM IWR-1 and with either vehicle (control), 25 ng/ml activin A, 1.5 μM CHIR99021 or both activin A and CHIR99021 (Fig. 1A). Treatment did not affect the efficiency of cell line derivation (Fig. 1B). There were few differences in morphology of ESC among treatments after 1 month of culture except cells cultured with activin A were less compact and colonies slightly more flattened than cells from the other treatments (Fig. 1C). Treatment with either activin A, CHIR99021 or both reduced amount of immunoreactive SOX2. Treatment with activin A alone increased immunoreactive NANOG while CHIR99021 decreased immunoreactivity (Fig. 1D).

Intensity of fluorescent signal was quantified for both pluripotency factors. Immunoreactive SOX2 was affected by activin A (*P*=0.0011), CHIR99021 (*P*<0.0001) and the interaction (*P*<0.0001). Intensity of labeling was decreased by all treatments as compared to control with the greatest decrease for cells treated with CHIR99021 or activin A and CHIR99021 (Fig. 1E). The fluorescent signal for NANOG was approximately one tenth of that for SOX2 in the control medium. Immunoreactive NANOG was affected by both activin A and CHIR99021 (*P*<0.0001), with activin A increasing immunofluorescent labeling and CHIR99021 causing a decrease (Fig. 1F). These results suggest that both activin A and canonical WNT signaling reduced SOX2 expression by derived bESC although activin A increased expression of NANOG. It remains to be determined whether changes in amounts of these pluripotency factors affects the ability for long-term self renewal or capacity for subsequent differentiation.

### Actions of activin A and activation of canonical WNT signaling on development of the blastocyst into different cell lineages

**Fig. 2.**
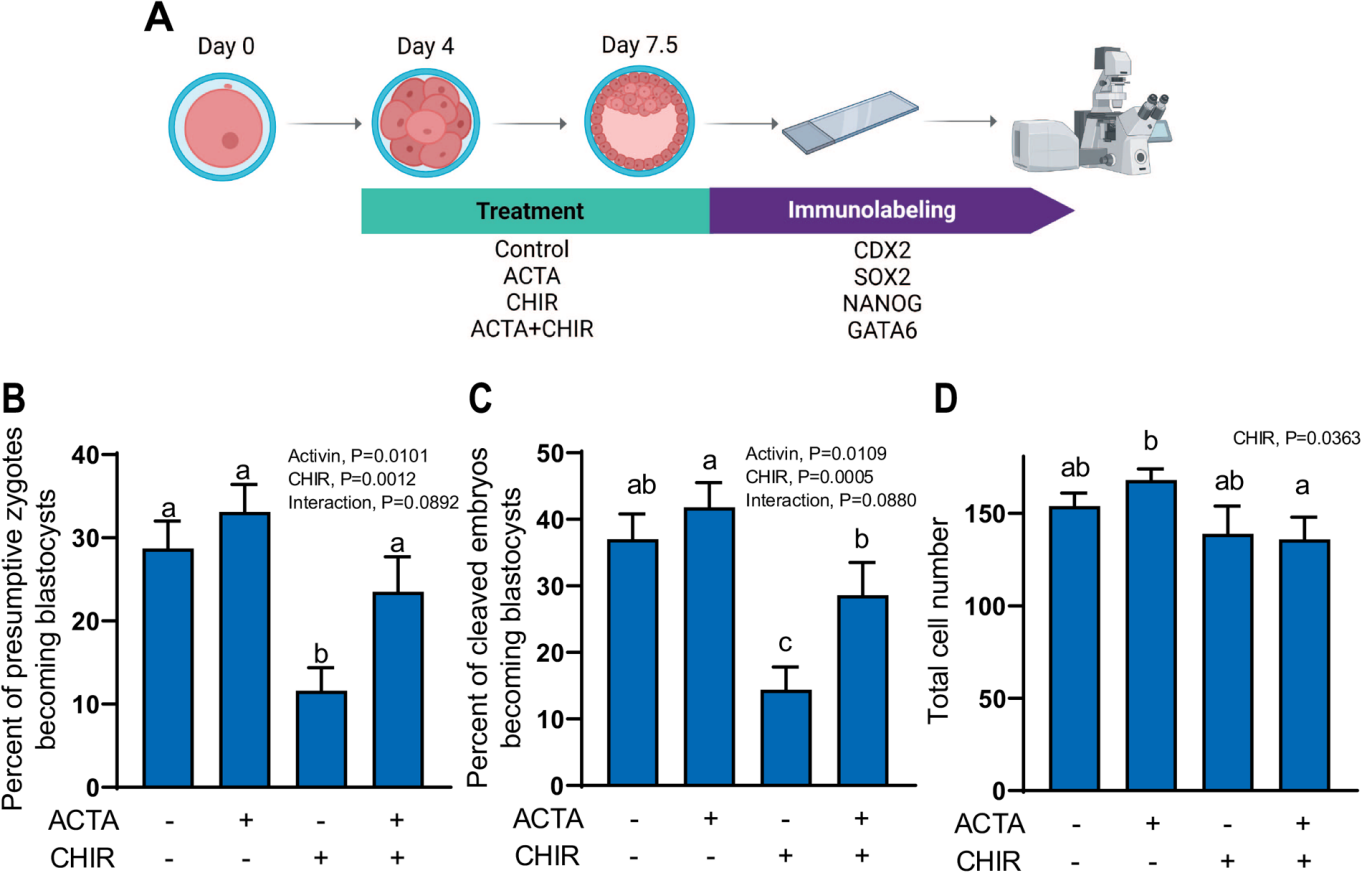
**Effects of activin A treatment and activation of canonical WNT on development of bovine embryos to the blastocyst stage.** (A) Experimental design. *In vitro* produced bovine embryos were treated with 25 ng/ml activin A (ACTA), 1.5 μM CHIR99021 (CHIR), 25 ng/ml ACTA+1.5 μM CHIR or control from day 4–7.5 of development. Blastocysts were collected for immunolabeling of lineage markers including SOX2, CDX2, NANOG and GATA6. Effects of treatments on percent of presumptive zygotes (B) and percent of cleaved embryos (C) becoming blastocysts were observed on day 7.5 in six embryo production replicates. (D) Total cell numbers of blastocysts. Data in panels B–D represent least-squares means±SEM. The *P*-value for main effects and the interaction that were *P*<0.10 or less are shown in the upper left regions of each figure. Bars with different letters differ (*P*<0.05) as determined by mean-separation test.

An additional experiment was conducted to evaluate whether activin A and CHIR99021 exerts similar effects on regulation of SOX2 and NANOG during the first and second lineage segregations. Embryos were cultured from day 4 to day 7.5 in the presence or absence of activin A and CHIR99021 (Fig. 2A). Percent of putative zygotes that cleaved (measured before treatment) was not affected by treatment (data not shown). Activin A had positive effects on blastocyst formation, whether expressed as the percent of putative zygotes (Fig. 2B; *P*=0.0101) or cleaved embryos becoming a blastocyst (Fig. 2C; *P*=0.0109). In contrast, CHIR99021 significantly decreased the proportion of putative zygotes (*P*=0.0012) or cleaved embryos (*P*=0.0005) becoming a blastocyst (Fig. 2B and 2C). CHIR99021 treatment also compromised development by reducing number of blastomeres (Fig. 2D; *P*=0.0363). Thus, activin A promoted competence of embryos to become a blastocyst while CHIR99021 not only reduced ability of an embryo to become a blastocyst but also resulted in blastocyst formation at smaller-than-normal cell number.

**Fig. 3.**
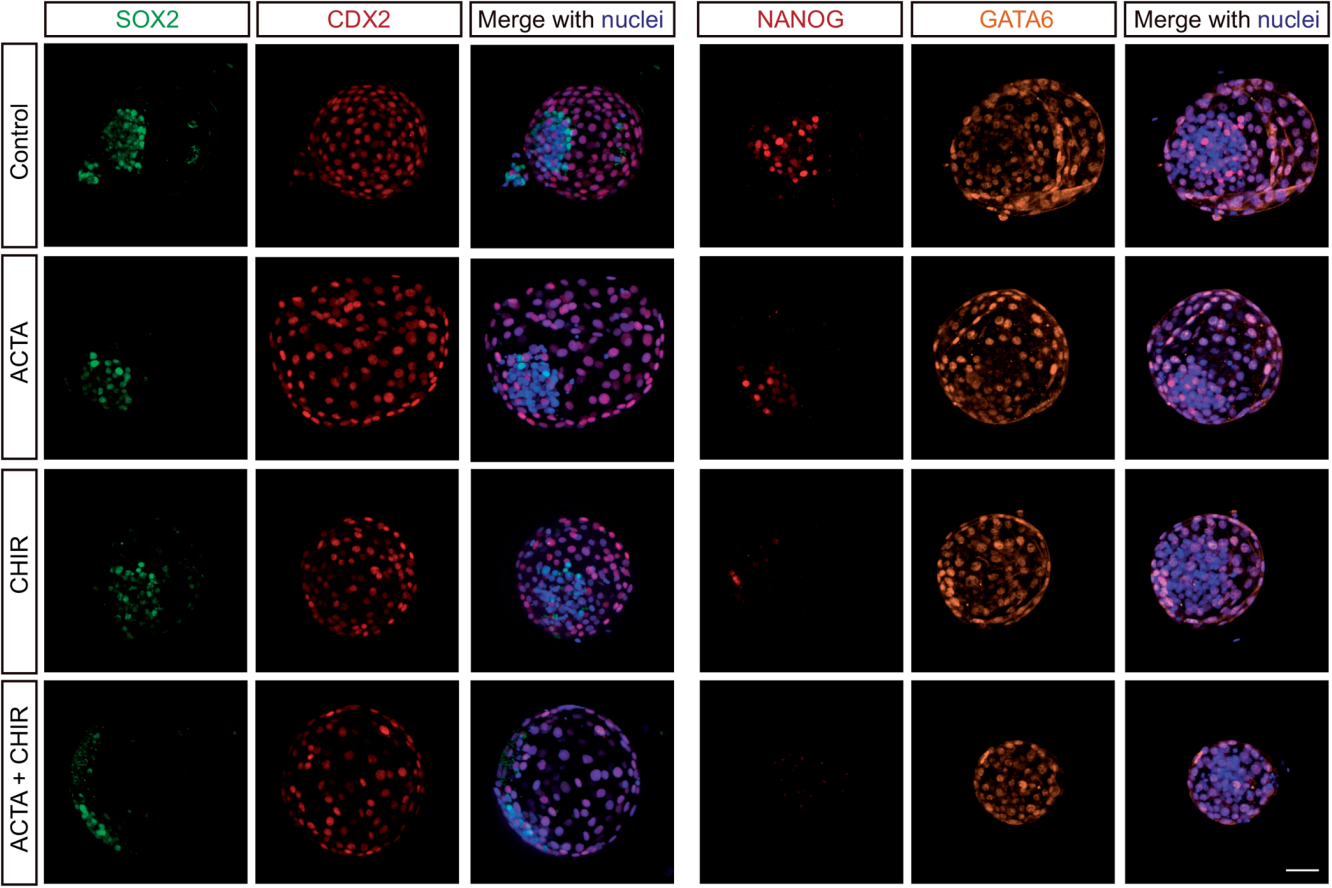
**Representative images of day 7.5 bovine blastocysts that were immunolabeled for SOX2 and CDX2 or NANOG and GATA6.** Shown are images generated by maximum projection of z-stacks. Scale bar: 50 µm.

**Fig. 4.**
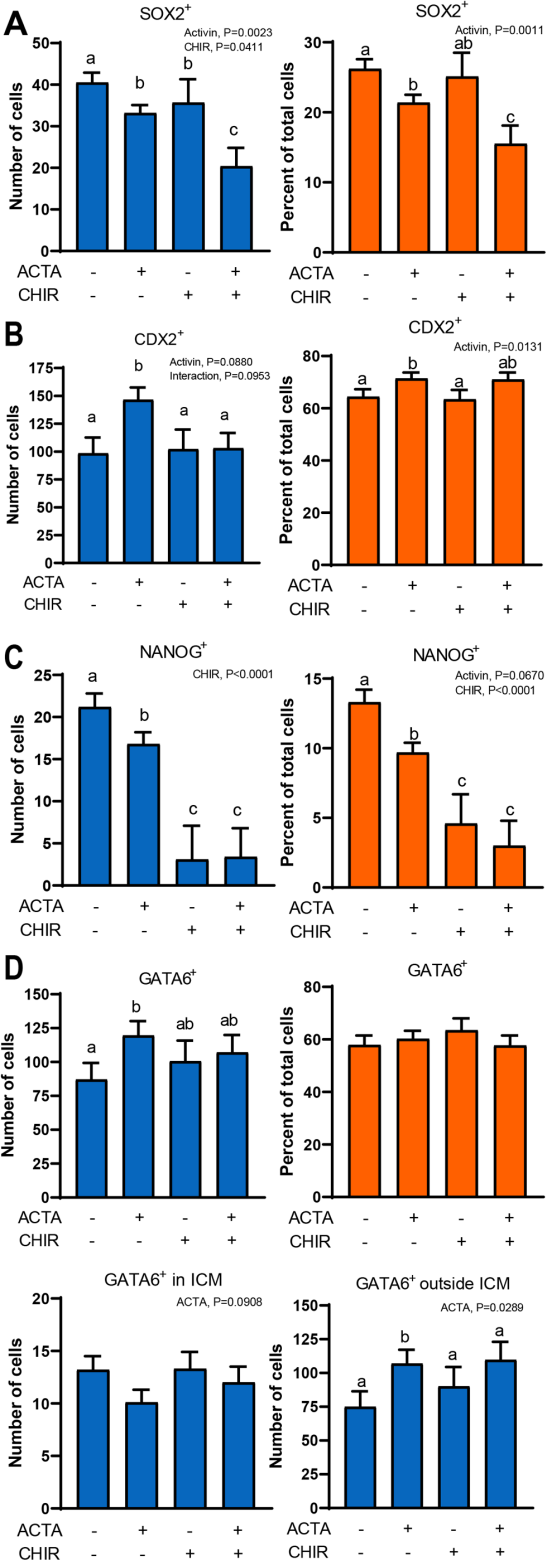
**Effects of activin A treatment and activation of canonical WNT signaling from day 4 to 7.5 of development on lineage segregation in the resultant blastocysts.** Treatments were 25 ng/ml activin A (ACTA), 1.5 μM CHIR99021 (CHIR), 25 ng/ml ACTA+1.5 μM CHIR or control from day 4–7.5 of development. Quantification of number and percent of cells that were SOX2^+^ (A), CDX2^+^ (B), NANOG^+^ (C), and GATA6^+^ (D). Data for numbers of GATA6^+^ cells were compiled for the entire blastocyst, the inner cell mass (ICM) only, and for cells outside the ICM. The *P*-values for main effects and the interaction that were *P*<0.10 or less are shown in the upper left regions of each figure. Bars with different letters differ (*P*<0.05) as determined by mean-separation test.

Representative images for immunolabeling for markers of ICM (SOX2), TE (CDX2), and epiblast (NANOG) are shown in Fig. 3 and results of quantitative analysis are shown in Fig. 4. Activin A decreased number of SOX2^+^ (*P*=0.0023) and NANOG^+^ cells (*P*<0.0001) but increased number of CDX2^+^ cells (*P*=0.0880). Similar results were found for percent of cells that were positive for SOX2 (*P*=0.0011), NANOG (*P*=0.0670) and CDX2 (*P*=0.0131). GATA6, which in the cow is expressed in both TE and hypoblast (Kuijk et al., 2012), tended to be reduced (*P*=0.0908) by activin A when examining positive cells in the ICM and was increased (*P*=0.0289) by activin A when examining positive cells outside the ICM. CHIR99021 reduced number of SOX2^+^ (*P*=0.0411) cells and number (*P*<0.0001) and percent (*P*<0.0001) of NANOG^+^ cells but had no effect on absolute or relative numbers of CDX2^+^ or number of GATA6^+^ cells. Results indicate that activin A acts on the embryo to promote the TE lineage and slightly inhibit ICM formation while activation of canonical WNT signaling attenuated formation of the ICM and epiblast lineages.

## DISCUSSION

These results indicate that activin A and canonical WNT signaling regulate expression of core pluripotency transcription factors in bESC and modify lineage segregation in the preimplantation bovine embryo. Activin A inhibited SOX2 expression and increased NANOG expression in bESC. In the embryo, activin had slight inhibitory effects on cells of the ICM as indicated by reductions in number of cells positive for SOX2, NANOG and GATA6. However, activin also increased the percent of embryos becoming blastocysts and the number of cells in the TE, namely those positive for CDX2 and those positive for GATA6 located outside the ICM. Activation of WNT signaling with CHIR99201 also reduced pluripotency markers as reflected by a decrease in expression of SOX2 and NANOG in bESC and in the number of blastomeres in the blastocyst that were positive for SOX2 and the number and percent of blastomeres positive for NANOG. Treatment with CHIR99201 also compromised blastocyst development. These latter results are consistent with other findings that inhibition of WNT signaling promotes development of ESC in the cow (Bogliotti et al., 2018; Xiao et al., 2021; Zhao et al., 2021) while activation of WNT signaling inhibits ability of embryos to become blastocysts (Denicol et al., 2013; Tríbulo et al., 2017). Thus, overactivation of WNT signaling can be inimical to development and maintenance of pluripotency in the cow.

The conditions tested for enhancing NANOG expression, addition of activin A and the WNT agonist CHIR99021, were based on a recent report where these two factors resulted in derivation of stable pluripotent pig ESC that expressed NANOG at a comparable level to other core pluripotency transcription factors (Choi et al., 2019). Activin A can enhance NANOG expression in the mouse (Vallier et al., 2009; Wu et al., 2015), human (Xu et al., 2008; Vallier et al., 2009) and pig (Yang et al., 2017c) and, as shown here, in bESC. Addition of activin/NODAL pathway inhibitor SB431542 reduced transcription of *NANOG* in pluripotent stem cells in the mouse (Wu et al., 2015), human (Xu et al., 2008) and pig (Choi et al., 2019). Thus, effects of activin signaling on NANOG expression in stem cells seem conserved among the mammals examined.

Treatment of bESC with CHIR99021, either in the presence or absence of activin A, reduced expression of SOX2 and NANOG by bESC. Furthermore, the WNT agonist also reduced number of SOX2^+^ cells and number and percent of blastomeres positive for NANOG in the blastocyst. The present study is thus in agreement with previous reports (Bogliotti et al., 2018; Choi et al., 2019; Gao et al., 2019; Vilarino et al., 2020; Soto et al., 2021; Xiao et al., 2021; Zhao et al., 2021) that support the notion that inhibition of canonical WNT signaling is essential for maintenance of the pluripotent state of ESC in several livestock species. A recent comprehensive analysis of signaling pathway modulation also was indicative that activation of canonical WNT signaling was associated with reduction in degree of stemness in both naïve and primed human ESC (Bayerl et al., 2021).

The conclusion that canonical WNT signaling is inhibitory to pluripotency in the cow must be tempered by the fact that effects of CHIR99021 are concentration dependent. Indeed, treatment with 0.3 µM CHIR99021 allowed maintenance of SOX2 and POU5F1 expression in bESC under a feeder-free condition in the absence of WNT inhibitor (Soto et al., 2021). In the same experiment, treatment with 3 µM caused loss of expression (Soto et al., 2021). In the mouse, in contrast, 3 µM CHIR99021 promoted *Nanog* transcript abundance in EpiSC and ESC (Wu et al., 2015; Ai et al., 2016). The underlying mechanisms responsible for concentration-dependent actions of WNT activation for stemness factor expression, including species variation and actions on ESC and associated feeder cells, remain to be determined. Similarly, actions of WNT signaling on development of the embryo to the blastocyst stage may also depend on the magnitude of the WNT signal. There are reports that WNT signaling can enhance the proportion of bovine embryos becoming a blastocyst (Aparicio et al., 2010) and increase NANOG^+^ cells in the blastocyst (Kuijk et al., 2012).

The increase in NANOG expression driven by activin signaling in bESC was associated with a decrease in SOX2 expression, which is in line with previous observations in mouse EpiSC and human ESC (Vallier et al., 2009; Chng et al., 2010). Although NANOG and SOX2 are both core pluripotency transcription factors, they have distinct functions in lineage specification in ESC (Wang et al., 2012). Gene knockdown experiments showed that NANOG suppresses neuroectoderm commitment in human ESC while SOX2 represses primitive streak differentiation (Wang et al., 2012). Given that activin A is a well-known endoderm inducer (Kubo et al., 2004; D'Amour et al., 2005), it is possible that addition of activin A primes bESC for an endoderm fate by reducing amounts of SOX2. It is also possible that the concentration of activin A used, 25 ng/ml, was too high to preserve pluripotency, especially in the presence of mouse embryonic fibroblasts, which presumably secrete activin A into the medium (Greber et al., 2010). Concentrations of activin A higher than 100 ng/ml (Vallier et al., 2009) can lead to expression of endoderm markers.

In other studies, loss of expression of SOX2 was associated with differentiation of bESC (Bogliotti et al., 2018; Soto et al., 2021; Xiao et al., 2021). It remains to be seen whether the downregulation of SOX2 by activin A affects pluripotency of bESC. Studies in primed pluripotent hESC indicated that knockdown of *SOX2* by short interfering RNA decreased expression of other pluripotency markers and induced differentiation towards TE and endoderm lineages (Fong et al., 2008; Adachi et al., 2010). However, ESC with low expression of SOX2 can retain pluripotency if expression of other pluripotency markers is retained (Wang et al., 2012).

Treatment of bovine embryos with activin A enhanced the proportion that became a blastocyst, which is consistent with previous observations in which activin A was added at day 5 of development (Trigal et al., 2011; Kannampuzha-Francis et al., 2017; Tríbulo et al., 2018). In contrast, treatment of bovine embryos with activin A from day 1 to day 3 reduced development to the blastocyst stage (Trigal et al., 2011), suggesting that action of this growth factor was dependent on the timing of treatment. Activin A also promoted expansion of CDX2^+^ cells (TE) and suppressed number and percent of cells that were SOX2^+^ (ICM) and percent of cells that were NANOG^+^ (epiblast). Given the role of TE cells in blastocoel formation (see review by Marikawa and Alarcón, 2009), enhancement of development to the blastocyst stage by activin A may be associated with its positive effects on the TE.

Similar actions of activin A to promote TE formation and inhibit epiblast were seen in the mouse embryo (Xiang et al., 2018), with effects at 500 and 3000 ng/ml but not at 100 ng/ml. Only one concentration of activin A was tested in the present experiment and it remains to be determined whether activin A exerts similar actions on cell lineages in the blastocyst at other concentrations.

The discrepancy between actions of activin A on the preimplantation embryo, in which numbers of cells for both SOX2 and NANOG were reduced, and bESC, in which activin A decreased SOX2 expression but increased NANOG expression, is probably a consequence of derivation of NANOG^+^ blastomeres in the blastocyst from SOX2^+^ blastomeres. NANOG is generally considered as an epiblast marker (Chambers et al., 2003; Silva et al., 2009) and is expressed later in development in the bovine embryo than SOX2 (Kuijk et al., 2012; Goissis and Cibelli, 2014; Wei et al., 2017). Activation of *NANOG* in the bovine embryo is blocked by disruption of *SOX2* expression (Goissis and Cibelli, 2014).

In conclusion, activin A and canonical WNT signaling regulate expression of core pluripotency transcription factors in bESC and modify lineage segregation in the preimplantation bovine embryo. In bESC, activin A inhibits SOX2 expression and increases NANOG expression. In the embryo, activin A promotes competence of the embryo to become a blastocyst, possibly because of its actions to increase formation of the TE. At least at the concentration tested, the growth factor also reduces formation of pluripotent blastomeres positive for SOX2 and NANOG. Activation of canonical WNT signaling with CHIR99201 caused a reduction in pluripotency markers in both bESC and the blastocyst, reinforcing the idea that over-activation of canonical WNT signaling suppresses pluripotency.

## MATERIALS AND METHODS

### Materials

Recombinant human activin A was from Peprotech (Rocky Hill, NJ, USA), recombinant human epidermal growth factor (EGF) was from Thermo Fisher (Bridgewater, NJ, USA), recombinant human fibroblast growth factor 2 (FGF2) was from Peprotech, and follicle stimulating hormone (FSH) was from Vetoquinol (Fort Worth, TX, USA). The tankyrase inhibitor IWR-1 was from Sigma-Aldrich (St. Louis, MO, USA), CHIR99021 was from Selleckchem (Houston, TX, USA) and the ROCK inhibitor Y-27632 was from Enzo (Farmingdale, NY, USA). Rabbit IgG monoclonal anti-human SOX2 (clone EP103) was from Biogenex (Fremont, CA, USA), mouse IgG1 monoclonal anti-human NANOG (clone hNanog.2) was from Thermo Fisher, mouse monoclonal anti-CDX2 (CDX2-88) was from Biogenex (Fremont, CA, USA), and rabbit IgG polyclonal anti-GATA6 (H-92) was from Santa Cruz Biotechnology (Dallas, TX, USA). Secondary antibodies (all cross-absorbed) were purchased from Thermo Fisher and were goat polyclonal IgG anti-rabbit IgG (H+L) coupled to Alexa Fluor 488 (catalog number A-11008), goat polyclonal IgG anti-mouse IgG (H+L) coupled to Alexa Fluor 488 (catalog number A-32731), goat polyclonal IgG anti-rabbit IgG (H+L) coupled to Alexa Fluor555 (catalog number A-21428) and goat polyclonal IgG anti-mouse IgG (H+L) coupled to Alexa Fluor 647 (catalog number A-21236). Other chemicals were purchased from Thermo Fisher or Sigma-Aldrich unless otherwise stated.

### Embryo production

Bovine embryos were produced *in vitro* as detailed elsewhere (Tríbulo et al., 2019). Briefly, cumulus-oocyte-complexes from bovine ovaries (*Bos taurus, B. indicus* or admixtures of the two genotypes) were matured for 22 h in Tissue Culture Medium 199 containing 10% (v/v) fetal bovine serum, 0.2 mM sodium pyruvate, 1% (v/v) alanyl-glutamine (GlutaMAX; Thermo Fisher), 50 ng/ml EGF, 5 µg/ml FSH, 100 units/ml penicillin and 0.1 mg/ml streptomycin. Matured oocytes were fertilized with a pool of spermatozoa from three bulls in *in vitro* fertilization-Tyrode albumin lactate pyruvate medium for 16–18 h. Cumulus cells were removed by hyaluronidase digestion and putative zygotes (i.e. oocytes exposed to sperm) were cultured in groups of up to 30 in 50 μl microdrops of synthetic oviduct fluid bovine embryo 2 medium. Microdrops were covered with mineral oil and embryos were incubated at 38.5˚C in an atmosphere of 5% (v/v) oxygen and 5% CO_2_ in a humidified atmosphere until day 7.5.

### Derivation and maintenance of ESC

Procedures followed the protocol for establishment of stable primed pluripotent bovine ESC lines (Bogliotti et al., 2018). Blastocysts (non-expanded, expanded, hatching and hatched) at day 7.5 of development (fertilization=day 0) were harvested from culture, rinsed in HEPES-TALP medium (Tríbulo et al., 2019) and subjected to zona pellucida removal by manual dissection with a pair of 30 ga needles. Each zona-pellucida-free blastocyst was individually transferred into a well that was plated 16∼36 h earlier with irradiated CF1 mouse embryonic fibroblasts (Thermo Fisher Scientific) mouse in Nunc™ 4-well dishes. The feeder cells were cultured in Dulbecco Modified Eagle Medium supplemented with 10% (v/v) fetal bovine serum, 1% (v/v) alanyl-glutamine and 1% (v/v) penicillin-streptomycin. On the day of seeding blastocysts, the feeder cells were rinsed with DPBS and the medium was replaced with 0.5 mL ESC culture medium consisting of a custom mTeSR1 medium without transforming growth factor-β (Ludwig et al., 2006; Wu et al., 2015; Bogliotti et al., 2018) supplemented with 20 ng/ml FGF2, 2.5 µM IWR-1, 100 units/ml penicillin and 0.1 mg/ml streptomycin. Each blastocyst used for ESC derivation was randomly assigned within blastocyst stage to receive one of four treatments. The treatments were ESC culture medium (control), and ESC culture medium containing 25 ng/mL activin A, 1.5 µM CHIR99021 or 25 ng/mL activin A and 1.5 µM CHIR99021. A total of 12–13 blastocysts were cultured for each treatment.

For all treatments, the ROCK inhibitor Y-27632 (10 µM) was added to ESC culture medium on the day of blastocyst seeding and replaced by medium without Y-27632 the day after. The ROCK inhibitor was also added for 24 h each time cells were passaged. At 24 h after seeding, blastocysts that had not attached to the bottom of the well were manually assisted to attach by positioning them using a 30-gauge needle. Culture medium was refreshed each day. Cells were passaged onto 24-well plates on day 7 after blastocyst seeding, with new feeder cells prepared a day in advance. Wells were rinsed with calcium and magnesium-free DPBS, treated with 1x TrypLE™ Select Enzyme (Thermo Fisher) for 2 to 5 min in an incubator to generate a single-cell suspension. Cells were centrifuged and resuspended to dilute cells 1:5 or 1:10 (v/v) and plated at 0.5 ml.

All cultures were passaged regardless of the observed emergence of outgrowth from the blastocyst. A second passage was performed 7 days later. Subsequently, passages were performed every 2–4 days depending on the degree of confluency. The ROCK inhibitor was added for 24 h each time cells were passaged. Cell lines were not authenticated or tested for contamination.

### Actions of activin A and CHIR99021 on differentiation of the blastocyst

Embryos were produced as described above and cultured in 45 µl SOF-B2 until day 4 after fertilization. At this time, treatments were added in a volume of 5 µl to achieve the desired final concentration. Treatments were activin A (25 ng/ml), CHIR99021 (1.5 µM), 25 ng/ml activin A and 1.5 µM CHIR99021, and vehicle [Dulbecco phosphate-buffered saline (DPBS) containing 1 mg/ml bovine serum albumin (BSA) and 0.05% DMSO]. Blastocysts were then harvested for analysis of cell lineages using two-color immunofluorescence for either CDX2 and SOX2 or NANOG and GATA6 as described below.

The experiment was replicated on six occasions. The number of observations for total cell number were as follows: control, *n*=67; ACTA, *n*=112; CHIR99021, *n*=19; ACTA+ CHIR99021, *n*=30. The number of observations for SOX2 were as follows: control, *n*=54; ACTA, *n*=93; CHIR99021, *n*=10; and ACTA+ CHIR99021, *n*=18. The number of observations for CDX2 were control, *n*=14; ACTA, *n*=27; CHIR99021, *n*=10; and ACTA+ CHIR99021, *n*=18. The number of observations for NANOG were control, *n*=53; ACTA, *n*=85; CHIR99021, *n*=9; and ACTA+ CHIR99021, *n*=12. The number of observations for GATA6 were control, *n*=13; ACTA, *n*=19; CHIR99021, *n*=9; and ACTA+ CHIR99021, *n*=12.

### Immunolabeling

Bovine ESC were seeded onto Nunc™ Lab-Tek™ II Chamber Slides™ (Thermo Fisher Scientific) that were plated with feeder cells a day before. Steps for immunofluorescence were as previously reported (Bogliotti et al., 2018). Cells were fixed in 4% (w/v) paraformaldehyde in DPBS for 15 min and incubated in blocking buffer [DPBS containing 3% (v/v) normal goat serum and 0.3% (v/v) Triton X-100] for 1 h. Primary antibody incubation was for 2 h at room temperature and secondary antibody was for 1 h at room temperature. The primary antibodies were rabbit IgG monoclonal anti-human SOX2 diluted 1:300 (v/v) and 1.7 µg/ml mouse IgG1 monoclonal anti-human NANOG. Alexa-labeled secondary antibodies were used at a concentration of 2 µg/ml in a solution containing Hoechst 33342 (10 µg/ml). Antibodies were diluted in blocking buffer. Cells were covered with a glass coverslip using anti-fade medium after rinsing in DPBS.

Immunolabeling of day 7.5 blastocysts involved rinsing in DPBS containing 0.2% (w/v) polyvinyl alcohol (PVA), fixation in DPBS containing 4% (w/v) paraformaldehyde, permeabilization for 30 min in 0.25% (v/v) Triton X-100 in PBS-PVA, and incubation in blocking buffer [DPBS containing 5% (w/v) BSA] for 1 h at room temperature. Antibodies were diluted in blocking buffer for incubation at 4°C overnight (primary antibodies) or 1 h at room temperature (secondary antibodies). Primary antibodies were used in pairs including rabbit IgG monoclonal anti-human SOX2 diluted 1:300 (v/v) and 1.7 µg/ml mouse IgG1 monoclonal anti-human NANOG, SOX2 and ready-to-use mouse monoclonal anti-CDX2, or NANOG and 1 µg/ml rabbit IgG polyclonal anti-GATA6. Alexa-labeled secondary antibodies were used at a concentration of 2 µg/ml in a solution containing Hoechst 33343 (10 µg/ml).

Images of immunolabeled blastocysts and cells were obtained using a spinning disc (Andor DSD2, Oxford Instruments, Tubney Woods, Abingdon, UK) confocal microscope (Axioobserver.Z1, Zeiss) controlled by Andor IQ3 software (Oxford Instruments). The microscope was equipped with a Plan-APOCHROMAT 20X/0.8 objective (Zeiss) and an Andor Zyla sCMOS camera (Oxford Instruments). Z-stack images were taken at 1 µm intervals. Uniform exposure times, light intensities, and gains were used to observe samples for an individual experiment.

### Image analysis

Image analysis was performed using ImageJ (Ver. 1.52a, Wayne Rasband, NIH, Bethesda, MD, USA). Cell counting was achieved by use of the multi-point tool and manual counting. Quantification of intensity of labeling for SOX2 and NANOG in cells was performed with maximum projections generated from z-stacks of the SOX2, NANOG and Hoechst channels. A cell colony was selected using the freehand tool the threshold feature and data from the Hoechst channel was used to select nuclei only. The selected areas were then applied to images for the SOX2 and NANOG channels to measure fluorescent intensity of nuclei. Intensity was corrected by subtracting local background signals. On average, 9 to 12 cell colonies or areas were analyzed for each cell line. The number of blastomeres positive for SOX2, CDX2, NANOG and GATA6 was determined by manual counting of cells using maximum projections generated from z-stacks of the channels for pertinent antibody and Hoechst labeling.

### Statistical analysis

Data were analyzed using programs of the Statistical Analysis System (Ver. 9.4, SAS Institute, Cary, NC). Effects of treatment on percent of embryos establishing cell lines was determined by logistic regression using the GLIMMIX procedure. Other variables were analyzed by analysis of variance using the GLM procedure. Data included the fluorescent intensity of immunolabeling (calculated for each bESC cell line), percent of embryos becoming blastocysts (calculated for each replicate), and number and percent of blastomeres (calculated for each blastocyst). The statistical model considered treatment and either cell line (bESC) or embryo production replicate as independent variables. Orthogonal contrasts were used to partition effects of treatment into main effects of activin, CHIR99021 and the interaction. In addition, the pdiff mean separation test was performed to determine means that differed at *P*<0.05.
